# The Role of Secretion Systems, Effectors, and Secondary Metabolites of Beneficial Rhizobacteria in Interactions With Plants and Microbes

**DOI:** 10.3389/fpls.2020.589416

**Published:** 2020-11-09

**Authors:** Miriam Lucke, Mario Gabriel Correa, Asaf Levy

**Affiliations:** Department of Plant Pathology and Microbiology, The Robert H. Smith Faculty of Agriculture, Food and Environment, The Hebrew University of Jerusalem, Rehovot, Israel

**Keywords:** beneficial bacteria, plant growth promoting bacteria, biocontrol agents, root nodulating bacteria, rhizosphere, effectors, secretion systems

## Abstract

Beneficial rhizobacteria dwell in plant roots and promote plant growth, development, and resistance to various stress types. In recent years there have been large-scale efforts to culture root-associated bacteria and sequence their genomes to uncover novel beneficial microbes. However, only a few strains of rhizobacteria from the large pool of soil microbes have been studied at the molecular level. This review focuses on the molecular basis underlying the phenotypes of three beneficial microbe groups; (1) plant-growth promoting rhizobacteria (PGPR), (2) root nodulating bacteria (RNB), and (3) biocontrol agents (BCAs). We focus on bacterial proteins and secondary metabolites that mediate known phenotypes within and around plants, and the mechanisms used to secrete these. We highlight the necessity for a better understanding of bacterial genes responsible for beneficial plant traits, which can be used for targeted gene-centered and molecule-centered discovery and deployment of novel beneficial rhizobacteria.

## Introduction

The term rhizosphere was first defined by Hiltner, who described it as the soil compartment influenced by the root (Hiltner, 1904). The rhizosphere differs from the surrounding bulk soil and the plant endophytic compartment in microbial diversity (Hacquard et al., 2015) and its members influence the release of root exudates. Root exudates are responsible for shaping the microbial community structure, including attraction of beneficial microbes (Clark, 1949; Zhalnina et al., 2018; Korenblum et al., 2020). After successfully colonizing plant roots, beneficial microbes secrete proteins and secondary metabolites, relevant for nutrient acquisition, improved plant fitness, and inhibition of pathogen colonization (Pieterse et al., 2014; Bakker et al., 2018; Yu et al., 2019a). Beneficial microbes are subdivided in a coarse manner into plant growth promoting rhizobacteria (PGPR), biocontrol agents (BCAs), and root-nodulating bacteria (RNB; Berendsen et al., 2012). PGPR directly or indirectly induce plant growth *via* secretion of secondary metabolites, which are in turn involved in plant hormone synthesis and nutrient acquisition from soil (Lugtenberg and Kamilova, 2009). RNB are also referred to as biofertilizers. They interact with legume roots as mutualists. Nodules allow the energetically expensive process of nitrogen fixation. The ammonia produced in the nodules as part of this process is transported into the plant cells in exchange for carbon required for bacterial growth. BCAs or biopesticides in the roots act by eliminating phytopathogens and pests, either indirectly by induction of the plant immune response through induced or acquired systemic resistance, or directly by producing and releasing antimicrobial and pesticidal toxins or by physical niche occupation (Kuc and Tuzun, 1992; Van Wees et al., 1997; Zamioudis and Pieterse, 2012; Bernal et al., 2018).

**Figure 1:**
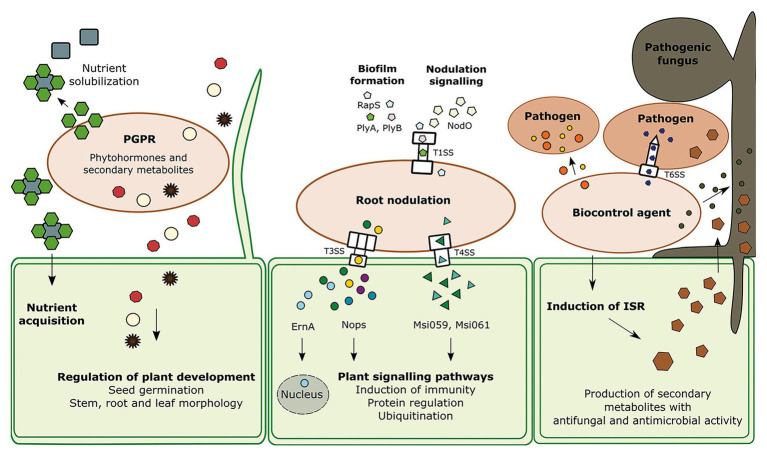
The interaction of the three groups of beneficial bacteria with other species; plant growth promoting rhizobacteria (PGPR), root nodulating bacteria (RNB), and biocontrol agents (BCAs) and their neighboring cells. PGPR produce various secondary metabolites including phytohormones which are regulating several processes in the plant development such as seed germination, stem, leaf, and root morphology. Another feature of PGPR is the solubilization of nutrients. RNB contain several secretion systems that can transport effectors directly into the host cell to regulate certain processes. Type I protein secretion system (TISS) of *Rhizobium leguminosarum bv. Viciae* is responsible for biofilm formation *via* the effectors PlyA, PlyB, and Rhizobium-adhering proteins (RapS). The T1SS is recognizable due to the outer membrane protein TolC. Type III secretion system (T3SS) and type IV secretion system (T4SS) are secreting effectors which can trigger protein regulation and induce plant immune responses. At least one effector travels into the plant cell nucleus. BCAs produce antibacterial and antifungal protein toxins and small molecules. Proteinaceous toxins are transferred through the Type VI secretion system (T6SS), a powerful nanoweapon, into the host cell. Specific antimicrobials can kill phytopathogens like fungi, oomycetes, and bacteria. In addition, biocontrol agents can trigger the plant immunity pathway induced systemic resistance (ISR), which leads to the production of antimicrobials which can eliminate a broad spectrum of organisms.

The effectiveness of beneficial microbes is frequently dependent on secretion systems. Some secretion systems allow translocation of proteins, called effectors, directly from one cell into another without being degraded or utilized by another organism. Other secretion systems and efflux pumps release proteins and secondary metabolites into the medium, respectively. The secreted proteins and metabolites play roles in root colonization, as well as in interactions with the plant immune response and the surrounding prokaryotic and eukaryotic organisms (Figure 1; Lugtenberg and Kamilova, 2009; Pieterse et al., 2014; Wu et al., 2018; Jamali et al., 2020). The goal of this minireview is to describe important bacterial secreted effectors, secondary metabolites and secretion systems which play a role in the interactions of beneficial microbes with plants and surrounding microbes, including bacteria and fungi.

## Plant Growth Promoting Rhizobacteria

Plant growth promoting rhizobacteria can improve the plant growth in multiple ways. They can indirectly promote growth by forming a biofilm that serves as a protective layer against pathogens or as an enhanced surface for nutrient acquisition from the surrounding soil (Weselowski et al., 2016). They can also produce and secrete growth phytohormones or their intermediates, which directly increase the root surface area, and promote plant development, growth and health (Figure 1; Spaepen et al., 2014). Additionally, PGPR increase abiotic stress tolerance in crops.

Plant growth promoting rhizobacteria secrete organic acids and other secondary metabolites that solubilize macronutrients and micronutrients and increase their bioavailability for plants. Nitrogen and phosphorus are two of the essential macronutrients for plant growth. Nitrogen fixation will be discussed in the next section. Phosphorus in soil is highly unavailable for plants. Beneficial microbes mobilize phosphorus *via* organic chelators like citric, gluconic and malic acid. The secretion of these acids leads to a decrease of the soil pH and production of plant bioavailable HPO_4_^2−^ (Rodríguez and Fraga, 1999; Ivanova et al., 2006; Yasmin et al., 2009). Some members of the *Bacillus*, *Pseudomonas* and *Enterobacter* genera are very efficient phosphorus solubilizing bacteria and have been shown to improve yield and growth of crops (Jha et al., 2012; Goswami et al., 2014).

Iron is an essential micronutrient for plant growth and development and iron-deficient plants suffer from yellow stripe chlorosis in young leaves (Abadía et al., 2002). The application of *Alcaligenes* 637Ca and *Staphylococcus* MFDCa1 to pear and apple roots, respectively, increased the foliar enzymatic activity of a plant enzyme that is responsible for Fe3+ reduction and helps plants uptake iron under iron deprivation (İpek et al., 2017; Aras et al., 2018). Some *Pseudomonas* spp. PGPR secrete siderophores like carboxylates, catecholate, and hydroxamate for Fe acquisition in *Zea mays*. Siderophores also have antimicrobial properties against the phytopathogens *Rhizoctonia solani* and *Sclerotium rolfs* (Sharma and Johri, 2003; Yu et al., 2011; Scagliola et al., 2016; Trapet et al., 2016; Mullins et al., 2019).

In contrast to the indirect effect of solubilization of nutrients on plant growth, phytohormone production by PGPR directly interfaces with plant growth signaling networks. The five main phytohormones are produced by PGPR: auxins, cytokinins, gibberellins (GAs), ethylene (ET), and abscisic acid. One of the most studied PGPRs is the *Azospirillum* genus. *Azospirillum. brasilense* consists of four different pathways to produce IAA, which is the most common phytohormone from the auxin class. The indole-3-pyruvate pathway produces the highest amount of IAA (Kloepper et al., 1989; Puente et al., 2004). The IAA biosynthesis pathways are well understood in bacteria, but the reason for the existence of those pathways in bacteria is still unknown (Molina et al., 2018). The indole-3-pyruvate pathway is highly dependent on the key enzyme indole-3-pyruvate decarboxylase encoded by the gene *ipdC* (Spaepen et al., 2014). *ipdC* alone is responsible for induction of root hair formation as was shown by laboratory inoculation experiments of *Arabidopsis thaliana* with *A. brasilense* SP245 strain (Cohen et al., 2008; Rivera et al., 2018). However, field experiments are inconsistent and did not reproduce the results (Díaz-Zorita and Fernández-Canigia, 2009). Generally, field experiments inoculated with *Azospirillum* showed an inconsistent increase in grain yield (Dobbelaere et al., 1999; Vande Broek et al., 1999). *Azospirillum* has many features, in addition to auxin production, which could lead to plant growth promotion including nitrogen fixation, siderophore production, and phosphate solubilization. Hence, there might be a growth promotion as an additive or a synergistic combination of the various pathways (Spaepen et al., 2008). The technological progress in the field allows us to combine large-scale ecological studies with a reductionist genetic approach that reveals bacterial genes that promote growth. A recent study using a 185-member bacterial community showed that this community causes *Arabidopsis* root growth inhibition. Interestingly, several strains of the *Variovorax* genus were found to maintain root development. Further genetic approach identified that the *Variovorax* genomes encode an auxin degrading operon that is necessary and sufficient for causing this beneficial phenotype even in such a complex ecosystem (Finkel et al., 2020).

Another example for phytohormone production in rhizobacteria is of the phytohormones gibberellins. GAs are involved in many developmental processes in plants, such as flowering regulation, seed germination, stem and leaf elongation, and pollen maturation (Achard et al., 2007; Ariizumi and Steber, 2007). Biosynthesis of GA was found in many bacteria such as *Bacillus pumilus, Bacillus licheniformis*, and *Leifsonia soli* (Kang et al., 2016; Kim et al., 2017). *Leifsonia soli* SE134 has been shown to enhance plant growth of the GA deficient *Waito-C* rice dwarf mutant cultivar and can extend shoot length, plant weight, and seed germination in cucumber, and tomato under greenhouse conditions, which may be due to GA synthesis (Kang et al., 2014).

Cytokinins are another group of growth-stimulating phytohormones that are responsible for cell division, plant senescence, seed germination, flower and fruit development, and apical dormancy (Akhtar et al., 2020). *Pseudomonas fluorescens* G20-18 and 6–8 strains produces cytokinins (García de Salamone et al., 2001; Pallai et al., 2012). It has been shown that canola inoculated with G20-18 and 6–8 strains had greater root length than the non-inoculated control in a gnotobiotic assay (Pallai et al., 2012). Großkinsky et al. (2016) revealed by constructing various knock-out and gain of function mutants of G20-18, that cytokinins have a protective role against *Pseudomonas syringae* pv. tomato DC3000 and can suppress disease response in *A. thaliana* (Großkinsky et al., 2016).

The aforementioned studies showed that PGPR can secrete multiple molecules which lead to various phenotypes. Exactly which conditions favor release of these beneficial growth-promoting molecules is still poorly understood. Knowing these conditions is important given that abiotic and biotic stressors can affect phytohormone production (Díaz-Zorita and Fernández-Canigia, 2009).

Ethylene is an important plant growth hormone that ameliorates harmful effects of abiotic stress conditions in plants (Glick, 2014). Its precursor is 1-aminocyclopropane-1-carboxylate (ACC). PGPR can improve plant tolerance to abiotic stressors *via* the production of ACC deaminase, which cleaves ET and produces α-ketobutyrate and ammonia. ACC deaminase indirectly counteract saline plant growth inhibitory effects in plants, hence plants are more salt stress tolerant (Orozco-Mosqueda et al., 2019, 2020). *Pseudomonas putida* UW4 carrying the *acdS* gene that encode for ACC deaminase was able to restore 66% of canola shoot fresh mass when grown in cold temperatures under high salt levels. Remarkably, the Δ*acdS* strain yielded only 20% of shoot fresh mass under the same conditions, demonstrating the importance of this single bacterial gene in promoting plant growth (Cheng et al., 2007). Trehalose, a non-reducing disaccharide, is especially induced in bacteria under saline stress and reduces osmotic, ionic and saline stress responses, by interacting with ABA, volatile compounds and exopolysaccharides (EPS; Avonce et al., 2006). Recently the synergetic effect of trehalose accumulation and ACC-deaminase production has been discovered in *Pseudomonas* sp. UW4 protecting tomato plants under saline stress. The plants were unable to survive the abiotic stress when the UW4 *acdS* and *treS* (trehalose synthesis) genes where knocked out (Orozco-Mosqueda et al., 2019). More detailed information about the synergistic effect of rhizobacteria produced ACC deaminase and plant compounds were reviewed recently by Forni et al. (2017). ACC deaminase also plays a role in synergetic function with other soil living-organisms such as in rhizobacteria for induced nodulation. *Pseudomonas fluorescens* YsS6 promotes the growth of *Rhizobia tropici* CIAT899, leading to an induced growth of *Phaseolus vulgaris*. The plant growth induction was only observed when YsS6 expressed *acdS* (Nascimento et al., 2019).

## Root-Nodulating Bacteria

Root-nodulating bacteria have developed an impressive and complex symbiosis with their legume host. One of the first steps in this relationship, is the secretion of flavonoids by the host plant that diffuse across the membrane of the Rhizobia and induce synthesis of the NodD protein which activates transcription of other genes involved in nodulation including Nod factor (NF) production (Wang et al., 2012). NFs are primary signal molecules produced by bacteria and detected by the plant to induce nodule organogenesis (Nelson and Sadowsky, 2015). In addition to NFs, other molecules and proteins mediate other aspects of the rhizobia-legume symbiosis such as root colonization, symbiont recognition and suppression of the plant immune system. To perform all of these tasks, Rhizobia make use of special secretion systems that translocate effectors to their host. These include type I, type III and IV secretion systems (Cianciotto, 2005; Schmeisser et al., 2009; Nelson and Sadowsky, 2015).

Type I protein secretion system (TISS) of *Rhizobium leguminosarum bv. viciae* is encoded by the *prsD* and *prsE* genes. This T1SS is responsible for secretion of the EPS-glycanases PlyA and PlyB (Russo et al., 2006). These enzymes play a key role in biofilm formation; by cleaving the EPS chains they modulate the structure and maturation of the biofilm. Mutations in *prsD* and *prsE* greatly suppress the formation of biofilm on glass surfaces (Russo et al., 2006). Biofilm formation is an important step in root colonization and in symbiotic interaction formation. Once rhizobia attach to root hairs, they aggregate and form a biofilm, which is encased in a structure called a cap that is made of cellulose and EPS (Smit et al., 1987; Downie, 2010). Some proteins such as Rhizobium-adhering proteins (Raps) are required for stability of the cap, and are exported through the PrsDE T1SS (Smit et al., 1987; Russo et al., 2006; Krehenbrink and Allan, 2008; Poole et al., 2018). RapA1 is a calcium-binding Rap located at the cell pole (Poole et al., 2018). RapA1 overexpression in *R. leguminosarum bv. trifolii* R200 increased attachment to red clover roots by up to 5-fold and its overexpression in *Rhizobium etli* enhanced the capability of attachment to common bean roots (Mongiardini et al., 2009; Frederix et al., 2014). TISS also secretes NodO, a well-studied protein from *R. leguminosarum*, that is critical for signaling during nodulation (Finnie et al., 1997).

TolC is an integral membrane protein that is part of the outer membrane component of T1SS. TolC from *Sinorhizobium meliloti* functions in the symbiotic relationship with *Medicago sativa* (Cosme et al., 2008). *S. meliloti tolC* mutant showed an 8-fold reduction in the number of nodules compared with the wild type and presented an ineffective nitrogen fixation in the roots of *M. sativa* (Cosme et al., 2008). TolC may participate in the efflux of antimicrobial compounds produced by the host plant, resistance to osmotic or oxidative stress, polysaccharide biosynthesis, and the secretion of proteins or other molecules relevant for the symbiosis, such as NFs, that can affect directly or indirectly the formation of nodules in the roots of *M. sativa* (Srinivasan et al., 2015; Mergaert, 2018).

Other secretion systems, such as the type III secretion system (T3SS), are employed for effector translocation into the host plants. T3SS is mostly studied for its role in plant disease. The effectors can interfere with plant signaling and plant cell recognition. Transcriptional studies have shown expression of T3SS genes at different stages of the Plant-*Rhizobium* interaction such as root colonization, infection and nodulation. The T3SS of *Bradyrhizobium japonicum* USDA110 is expressed in infection threads and developing nodules of soybean (Zehner et al., 2008). Several T3SS genes of *Rhizobium* sp. *NGR234* are expressed in mature nodules of *Cajanus cajan* and *Vigna unguiculate* (Viprey et al., 1998; Perret et al., 1999; Tampakaki, 2014). Regulatory analyses of the T3SS of *Rhizobium* sp. *NGR234* showed that it is activated after Nod factors generation and its activity continues for at least 24 h (Kobayashi et al., 2004; Marie et al., 2004). These results indicate that effector secretion through T3SS concurs with development of the infection thread. T3SS is strongly regulated after sensing potential plant hosts.

T3SS genes called rhc (*Rhizobium* conserved), encode different nodulation outer proteins (Nops) that can be divided into two groups. The first group is composed of the core components of T3SS pilus that spans the plant cell wall (Saad et al., 2008; Deakin and Broughton, 2009; López-Baena et al., 2016). NopA and NopB are the major and minor subunits, respectively. NopX likely polymerizes to form a transmembrane pore (the translocon) through which other effectors enter the plant cytoplasm (Deakin and Broughton, 2009; López-Baena et al., 2016). The second group is composed of the effectors that are injected through T3SS machinery into the host cytoplasm. Several Sinorhizobial proteins secreted through the T3SS have been identified. These include NopL and NopP that may interfere with plant signaling pathways, as both can be phosphorylated by plant kinases and have shown to be responsible for optimal nodulation of host plants *Flemingia congesta* and *Tephrosia vogelii* (Bartsev et al., 2004; Skorpil et al., 2005; Gourion et al., 2015). NopL was shown to interfere with mitogen-activated protein kinase (MAPK) that is involved in pathogen recognition in both basal plant defense and R-mediated resistance (Pedley and Martin, 2005; Zhang et al., 2011). NopM belongs to the IpaH-SspH-YopM family of effectors found in animal pathogens, which are known to be involved in targeting nuclei of host cells and ubiquitination process (Bartsev et al., 2004; Skorpil et al., 2005; Rohde et al., 2007). A later study indicated a possible role for NopM as a functional E3 ubiquitin ligase domain in *Rhizobium* sp. strain NG234 (Xin et al., 2012). In the same study it was further mentioned that when expressed in *Nicotiana benthamiana*, NopM reduced reactive oxygen species (ROS) and induced plant defense gene expression (Xin et al., 2012). NopT effector has homology with the avirulence protein AvrPphB of the phytopathogen *P. syringae* and YopT of *Yersinia* spp. which are known to possess a protease activity. NopT mutants of NGR234 affected nodulation either positively (*P. vulgaris* cv. Yudou No. 1; *T. vogelii*) or negatively (*Crotalaria juncea*; Dai et al., 2008). NopM and NopT have shown to have either negative or positive effects in nodulation in a host dependent manner (Dai et al., 2008; Kambara et al., 2009)

Another effector, NopD in *Sinorhizobium fredii* HH103, has been predicted to be a C48 cysteine peptidase (Rodrigues et al., 2007). The C48 cysteine peptidase family contains the protein XopD, a T3SS effector from the plant pathogen *Xanthomonas campestris* (Hotson et al., 2003). It functions *in planta* to target SUMO-conjugated proteins (Hotson et al., 2003). XopD interferes with the plant’s ability to regulate the expression of specific proteins (Nelson and Sadowsky, 2015). NopC is a T3SS-dependent effector that lacks homologues in pathogenic bacteria but its function in plants is still unknown (Jiménez-Guerrero et al., 2015). NopJ acts as acetyltransferase that prevents phosphorylation of MAP kinases by acetylating the phosphorylation sites, thereby inactivating the MAP kinases (Mukherjee et al., 2006). Recently, a conserved T3SS effector, ErnA, was described in *Bradyrhizobium* (Teulet et al., 2019). Interestingly, this effector is targeted to the plant nucleus and may bind nucleic acids in the plant nuclei. Gain and loss of function experiments demonstrated the direct involvement of ErnA for nodule formation. All T3SS effectors and their predicted function are described in Table 1.

**Table 1:** Summary of all discussed bacteria, predicted function, and secreted molecules in this review. Some molecules are secreted from different bacteria.

Bacterial strain	Molecules	Predicted function	References
**Plant growth promoting**			
*Enterobacter, Bacillus, Pseudomonas*	Organic acids	Phosphate solubilization	Jha et al., 2012; Goswami et al., 2014
*Pseudomonas* spp. GRP3A, PRS9, *Pseudomonas chlororaphis* ATCC 9446	Siderophores	Fe acquisition	Sharma and Johri, 2003; Trapet et al., 2016
*Azospirillum brasilense* SP245	IAA production	Induction of root hair formation	Cohen et al., 2008; Molina et al., 2018
*Leifsonia soli* SE	Gibberellin	Induction of plant growth and seed germination	Kang et al., 2014
*Pseudomonas fluorescens* G20-18	Cytokinins	Suppression of disease resistance, cell elongation	Großkinsky et al., 2016
**Root nodulation**			
*Rhizobium leguminosarum bv. viciae* A34	Exopolysaccharide (EPS)-glycanases PlyA and PlyB	Biofilm maturation	Russo et al., 2006; Bogino et al., 2013
*Sinorhizobium meliloti*	TolC protein	Nodules production	Cosme et al., 2008; Srinivasan et al., 2015; Mergaert, 2018
*Rhizobium leguminosarum bv. trifolii* R200*, Rhizobium etli*	RapA1	Biofilm formation	Mongiardini et al., 2009; Ho et al., 2014; Poole et al., 2018
*Rhizobium leguminosarum* spp.	NodO	Signaling for nodulation	Finnie et al., 1997; Krehenbrink and Allan, 2008
*Sinorhizobium fredii* HH103	NopD	Regulating expression of plant proteins	Hubber et al., 2004; Rodrigues et al., 2007; Nelson and Sadowsky, 2015
*Bradyrhizobium japonicum* USDA110*, Sinorhizobium fredii* NGR234, HH103, USDA257	NopL	Induction of plant immune response	Pedley and Martin, 2005; Zhang et al., 2011
*Bradyrhizobium japonicum* USDA110*, Sinorhizobium fredii* NGR234, HH103	NopM	Ubiquitination process	Rohde et al., 2007; Burkinshaw and Strynadka, 2014; Zheng and Shabek, 2017
*Rhizobium etli* CNPAF512, *Sinorhizobium fredii* NGR234, HH103, USDA257	NopP	Phosphorylated by plant kinases	Bartsev et al., 2004; Skorpil et al., 2005; Gourion et al., 2015
*Sinorhizobium fredii* NGR234	NopT	Cysteine protease activity	Dai et al., 2008; Kambara et al., 2009; Gourion et al., 2015; Nelson and Sadowsky, 2015
*Rhizobium* sp. NGR234	NopJ	Inactivates MAP kinases	Mukherjee et al., 2006; Kambara et al., 2009; Gourion et al., 2015
*Mesorhizobium loti* R7A	Msi059	Regulating expression of plant proteins	Rodrigues et al., 2007; Nelson and Sadowsky, 2015
*Mesorhizobium loti* R7A	Msi061	Protein degradation of VirE2 and Vip1	Nelson and Sadowsky, 2015
*Bradyrhizobium strain ORS3257*	ErnA	An unknown function in the plant nucleus	Teulet et al., 2019
**Biocontrol**			
*Pseudomonas* spp., *Bacillus* spp.	Antibiotics	Virulence against phytopathogens	Guilleroux and Osbourn, 2004; Daval et al., 2011; Cao et al., 2018
*Pseudomonas fluorescens* Pf29Apr	DAPG	Downregulation of pathogenic enzymes	Daval et al., 2011
*Pseudomonas fluorescens* MFE01	T6SS related- toxins	Virulence against phytopathogens	Decoin et al., 2014
*Pseudomonas brassocaecearum* Q8r1*–*96	RopAA, RopB, RopM, DAPG	Induction of plant immune responses	Mavrodi et al., 2011
*Bacillus subtilis* BBG111	Cyclic lipopeptides (CLCPs)	Induction of plant immune responses	Ongena et al., 2005; García-Gutiérrez et al., 2013; Farace et al., 2015
*Bacillus velezensis*	Lipopeptide compounds	Antifungal	Cao et al., 2018

The bacterial type IV secretion systems (T4SS) is a unique system in its ability to transfer large nucleic acid molecules, in addition to proteins, across the cell envelope (Christie and Cascales, 2005; Sgro et al., 2019). Rhizobial T4SS shares strong homology to the VirB/VirD4 subunits found in *Agrobacterium* (Sullivan et al., 2002; Christie et al., 2014). The T4SS in *Agrobacterium tumefaciens*, is used for translocation of both T-DNA and effector proteins (Kuldau et al., 1990; Zupan and Zambryski, 1995). T4SS has been identified in rhizobia such as *Mesorhizobium loti* R7A (Hubber et al., 2007; Miwa and Okazaki, 2017) and *R. etli* CFN42 (Lacroix and Citovsky, 2016). T4SS could be involved in the nodulation process in *Rhizobium* in early stages. *M. loti* T4SS mutants delayed nodulation on *Lotus corniculatus* and allows effective nodulation on *Leucaena leucocephala* (Hubber et al., 2004, 2007). *R. etli* encodes a T4SS locus (*vir*) and is able to mediate transfer and integration of DNA into plant cell genome when provided with a T-DNA (Lacroix and Citovsky, 2016). However, a T-DNA-like sequences in *R*. *etli* was not identified, suggesting that *Rhizobium*-mediated plant transformation does not occur in nature, although it cannot be ruled out that other *Rhizobium* strains, not yet sequenced, harbor a T-DNA.

Thus far, only two T4SS candidate effector proteins were identified in rhizobia. These are Msi059 and Msi061 from *M. loti* R7A (Nelson and Sadowsky, 2015). Msi059 shares a partial protein sequence similarity to the XopD C48 cysteine peptidase (Rodrigues et al., 2007; Nelson and Sadowsky, 2015). The other T4SS effector Msi061, shares protein similarity with *A. tumefaciens* effector VirF (Tzfira et al., 2004). VirF interacts with the host Skp1 protein to facilitate protein degradation of effector proteins VirE2 and Vip1 leading to unbinding of the T-DNA after entry into the host cell (Tzfira et al., 2004). The specific role of the Msi059 and Msi061 in RNB remains unidentified, but the latest evidence suggests that they modulate protein expression levels *in planta* (Nelson and Sadowsky, 2015).

Type VI Secretion System (T6SS) contractile nanoweapons allows bacteria to inject toxins directly into prey cell membranes, periplasm or cytoplasm, leading to cell growth arrest. In rhizobia, T6SS sequence have been found in several species such as *R. leguminosarum*, *B. japonicum*, *M. loti*, *Sinorhizobium saheli*, and *S. fredii* (Bladergroen et al., 2003). T6SS was related to the prevention of nodulation on *Pisum sativum cv. Rondo* (Bladergroen et al., 2003). Recently, it was reported that *R. etli* Mim1 T6SS mutant produced plants with lower dry weight and smaller nodules than the wild-type strain, suggesting for the first time a positive role of T6SS in Rhizobium-legume symbiosis (Salinero-Lanzarote et al., 2019). The rhizobacterium *Azorhizobium caulinodans* ORS571 utilizes its T6SS to outcompete other strains during infection of its host *Sesbania rostrata* (Lin et al., 2018). However, the researchers could not show involvement in inter-bacterial competition *in vitro*. The nitrogen fixing bacteria *Azoarcus olearius* BH72 encodes two T6SS operons, one of which is strongly up-regulated when nitrogen is absent (Jiang et al., 2019). *Kosakonia* strains are endophytic nitrogen fixers involved in plant growth promotion in rice (Bertani et al., 2016). T6SS of *Kosakonia* KO348 is important for rhizoplane and endosphere colonization but it is not clear exactly how (Mosquito et al., 2019). One possibility is that the microbes use the T6SS to facilitate colonization by inhibiting competitors in the rhizosphere.

Although different secretion systems and effectors have been identified in RNB, their specific role in symbiosis and nodulation is still unclear. Further molecular and biochemical work should be done to characterize the molecular mechanisms leading to secretion of proteins and other molecules and their effects *in planta*.

## Biocontrol Agents

Biocontrol agents secrete a broad spectrum of secondary metabolites and proteins which can serve as antibacterial and antifungal compounds, such as enzymes which are able to degrade different compartments of various organisms (Mullins et al., 2019; Vesga et al., 2020). Some BCAs employ secretion systems to penetrate the neighboring cells and inject toxins into them. *Pseudomonas* spp., and *Bacillus* spp. are two of the most studied organisms in the BCA field. The most important and most studied secondary metabolites are antibiotics such as Phenazines, Phloroglucinols, Dialkylresorcinols, Pyrolnitrin, Pyoluteorin, Mupirocin, Peptide antibiotics, Hyrdogen cyanide, Rhizoxins, and Oxyvinylglycines (Raaijmakers et al., 2002; Weller, 2007; Mavrodi et al., 2011). *Bacillus velezensis* strains isolated from tomato rhizosphere strongly inhibit growth of *Ralstonia solanacearum* and *Fusarium oxysporum* under both laboratory and greenhouse conditions (Cao et al., 2018). This is done by production of different lipopeptide compounds whose production is stimulated during the BCA interaction with *R. solanacearum*. Recently, a survey of bacteria isolated from the phyllosphere of *A. thaliana* revealed novel antibiotics, with possible novel modes of actions (Helfrich et al., 2018). Antibiotics can be identified *via* HPLC and then tested for their antagonistic effect against different pathogens (Shahid et al., 2017). Many toxins (proteins or secondary metabolites) which are produced by beneficial bacteria have been studied beyond their antimicrobial/antifungal activity. For example, the BCA *P. fluorescens* Pf29Arp downregulates relevant pathogenicity enzymes (laccasses, exogluanases, and mitogen-activated kinases) in the fungus *Gaeumannomyces graminis var. tritici*, the causing agent of take-all disease (Guilleroux and Osbourn, 2004; Daval et al., 2011). Other assays can be used for profiling secondary metabolites, such as the use of LC-MS on crude extracts from BCA strains, *in silico* screening of antagonistic potential on pathogenic genes, and finally *in vitro* screening against specific pathogens (Jinal and Amaresan, 2020). *Burkholderia ambifaria*, a biocontrol agent was screened for its antimicrobial metabolites which led to detection of Cepacin A *via* LC-MS. Mutants for Cepain A production in *B. ambifaria* have a significantly reduced inhibition activity against *Pythium ultimum* in a pea infection model (Mullins et al., 2019).

Disease-suppressive soils prevent establishment of pathogens or lead to minor plant disease. The Raaijmakers group was able to demonstrate the involvement of beneficial bacteria from Burkholderiaceae family in disease-suppressive activity against *R. solani* (Chapelle et al., 2016; Carrión et al., 2018). They isolated representative Burkholderiaceae strains and uncovered genes involved in *in vitro* and *in situ* antifungal activity *via* the production of sulfurous volatile compounds (Carrión et al., 2018). Recently, they showed that an endophytic consortium of *Chitinophaga* and *Flavobacterium* consistently inhibited *Rhizoctonia solani* infection (Carrión et al., 2019). Moreover, they showed that the fungal infection enriched the root metagenome for chitinase genes and candidate biosynthetic gene clusters that likely produce antifungals. Finally, site-directed mutagenesis revealed a new NRPS-PKS gene cluster from *Flavobacterium* that is essential for disease suppression by the consortium. This is a fine example of how years of research revealed first specific BCA strains and later on their molecular mechanism that underlies a reproducible root microbiome that mediated plant protection.

As discussed already in the root nodulation section, Gram-negative bacteria can be equipped with different secretion systems. The T6SS translocates toxins into the neighboring cells that are killed if they do not have the matching immunity protein (Hood et al., 2010). T6SS genes were found, for examples, in *P. fluorescens* strain MFE01. Different T6SS effectors are injected by this strain. However, those toxins are not virulent against eukaryotic cells, but against a broad spectrum of pathogenic bacteria (Decoin et al., 2014). Bernal and colleges identified in *P. putida* KT2440 three T6SS clusters and 10 T6SS effector-immunity pairs. One of the T6SS loci is responsible to bactericidal activity against phytopathogens *in vitro* and in *planta* on *N. benthamiana*, although the *in planta* effect was mild (Bernal et al., 2017).

Biocontrol agents can also induce plant responses by secreting secondary metabolites. Often this results in an induction of the plant immune response called induced systemic resistance (ISR), which is regulated by the plant hormones jasmonic acid (JA) and ET (Berendsen et al., 2012; Pieterse et al., 2014). ISR is a response which is known to be triggered by rhizobacteria and leads to secretion of antimicrobial secondary metabolites from plants (Pieterse et al., 2012; Yu et al., 2019b). *Pseudomonas fluorescens* Q8r1–96 contains T3SS effectors RopAA, RopB, and RopM. In *N. benthamiana* these effectors suppress two plant immune pathways after leaf infection with *P. syringae* DC3000; the hypersensitive response and the production of reactive oxygens species (Mavrodi et al., 2011). Q8r1-96 also produces DAPG, which suppresses the take-all disease in wheat (Brazelton et al., 2008; Mavrodi et al., 2011; Kwak et al., 2012; Yang et al., 2020). *Bacillus* spp. is a well-established ISR elicitor. *Bacillus subitilis* BBG111 releases cyclic lipopeptides (CLPs), which are magnifying the plant microbe-associated molecular patterns (MAMPs) triggered immunity (MTI). The MTI recognizes microbe derived compounds, such as flagellins, lipopolysaccharides, and chitin that trigger the ISR pathway in rice against *R. solani* (Chandler et al., 2015; Lastochkina et al., 2019). This induction of ISR does not necessarily lead to the resistance against one phytopathogen since it is not species-specific (Chandler et al., 2015). *Bacillus* spp. and *Pseudomonas* spp. increase ISR in different kinds of crops (tomato, melon, and bean) against different organisms including fungi, bacteria, and nematodes (Ongena et al., 2005; García-Gutiérrez et al., 2013; Farace et al., 2015). The induction of ISR is very powerful, however its broad-spectrum activity may lead to killing of beneficial bacteria.

Often the combination of both PGPR and BCA can ensure both plant protectiveness and growth induction. Both traits can be tested *in vitro* on specific media. Liu et al. (2017) screened 196 PGPR strains based on their disease suppression for broad-spectrum antagonistic activity. In a second screen selected strains were tested for PGPR traits *in vitro*. For example, nitrogen fixation was tested on nitrogen-free semisolid medium and phosphate solubilization on media with different phosphate sources. In advanced screens, the PGPR strains were tested in planta for biological control of multiple plant diseases and most of them significantly reduced at least two tested diseases. Gene encoding antimicrobials were predicted but have not been experimentally validated (Liu et al., 2017).

The root nodules are also sites of active antimicrobial production. *Brevibacillus brevis* is an accessory species which resides near dominant rhizobia species. An untargeted *in planta* metabolomics study of this strain led to identification of nonribosomal peptides, Britacidin and gramicidin. Sequencing of the strain’s genome led to assignment of these antimicrobials to their cognate biosynthetic gene clusters. It is yet unknown whether these antimicrobials are used in competition between the natural nodule microbiome or protect it from pathogen infection (Hansen et al., 2020).

## Discussion

Much research has been conducted regarding PGPR, BCA, and RNB and many effectors are known and are not mentioned in this review. Despite the knowledge of those secreted molecules, their functionality *in planta* remains unclear (Bai et al., 2020; Kumar and Dubey, 2020; Zhou et al., 2020). The importance of the RNB secretion system in nodule formation and symbiosis between rhizobia and legumes is known; however, direct interactions of effectors and plant proteins and the specific processes regulated by the effectors are not understood. Many hypotheses have been postulated but were not confirmed experimentally (Sachs et al., 2018). High-quality ecological studies revealed the function of specific rhizobacteria in protecting plants against bacterial, fungal, and oomycete pathogens but did not reveal the compounds responsible for this effect (Duran et al., 2018; Kwak et al., 2018). High-density transposon screens coupled with in planta phenotyping can uncover the genes responsible for these antagonistic phenotypes. Another approach that should be applied is systematic gain of function approach to uncover the secondary metabolome encoded by the biosynthetic gene clusters of beneficial rhizobacteria. This can be done by using large scale operon cloning, induction of operons in organisms such as *Escherichia coli*, and applying the lysates on plants to couple microbial metabolites with beneficial functions.

We believe that genetic, metagenomics, transcriptomics, proteomics (secretome), and metabolomics analyses should increase our knowledge about the effectors and small molecules injected by rhizobacteria into the host, nearby pathogens, or released into the surrounding soil (Levy et al., 2018a). Identification of the specific genes, proteins and molecules responsible for growth promotion and protection against pathogens will allow a more accurate identification of beneficial strains and engineering of plant supportive microbiomes. We think that the entire field will gain important basic and applied insights by moving from identification of beneficial strains through extensive phenotype screening toward molecule-centered or gene-centered phenotypic associations. Identification of new genes and molecules that underlie a beneficial phenotype will allow accurate discovery of novel beneficial strains based on their genetic and chemical features identified from metagenome and metabolome surveys. Downstream functional analysis such as random mutagenesis of beneficial microbes coupled with identification of phenotypes *in planta*, protein binding assays to identify the binding partners of effectors in plant cells, or cell-based assays to show translocation of effectors into plants could improve molecular understanding of beneficial bacterial interaction with plants. Specifically, very little is known on the interaction of proteins and small molecules from beneficial microbes with the different branches of the plant immune system.

In addition to the lack of functional studies revealing the molecular basis for a beneficial microbial phenotype in plants, the understanding of bacteria communities in soil is also very partially understood. Recently, more studies include synthetic communities, revealing that certain assemblies of rhizobacteria are having a positive influence on plant fitness and health (Finkel et al., 2017; Helfrich et al., 2018). Sequenced and annotated genomes of those bacterial communities are available, however functional analysis lags behind (Finkel et al., 2017; Helfrich et al., 2018; Levy et al., 2018a,b).

## Author Contributions

All authors listed have made a substantial, direct and intellectual contribution to the work, and approved it for publication.

### Conflict of Interest

The authors declare that the research was conducted in the absence of any commercial or financial relationships that could be construed as a potential conflict of interest.
